# Working Memory With Emotional Distraction in Monolingual and Bilingual Children

**DOI:** 10.3389/fpsyg.2018.01582

**Published:** 2018-08-28

**Authors:** Monika Janus, Ellen Bialystok

**Affiliations:** Department of Psychology, York University, Toronto, ON, Canada

**Keywords:** bilingualism, executive control, emotional regulation, working memory, *n*-back

## Abstract

Extensive work has demonstrated the benefits of bilingualism on executive functioning (EF) across the lifespan. Concurrently, other research has shown that EF is related to emotion regulation (ER), an ability that is integral to healthy socio-emotional development. However, no research to date has investigated whether bilingualism-related advantages in EF can also be found in emotional contexts. The current study examined the performance of 93 children who were 9-years old, about half of whom were bilingual, on the Emotional Face *N*-Back Task, an ER task used to assess the interference effect of emotional processing on working memory. Bilingual children were more accurate than monolingual children in both 1-back and 2-back conditions but were significantly slower than monolingual children on the 2-back condition. There were significant effects of emotional valence on reaction time, but these did not differ across language groups. These results confirm previous research showing better EF performance by bilinguals, but no differences in ER were found between language groups. Findings are discussed in the context of our current understanding of the ER literature with potential implications for previously unexplored differences between monolingual and bilingual children.

## Introduction

Flexible and effective emotion regulation (ER) is critical for healthy psychosocial adjustment throughout development (Cole and Deater-Deckert, 2009; Eisenberg et al., 2010). The inability to properly regulate emotions, or emotional dysregulation, has been shown to underlie a range of maladaptive outcomes including aggressive behavior problems (e.g., Stieben et al., 2007; Lewis et al., 2008; Holley et al., 2017) and academic underachievement (e.g., Hawkins et al., 1999; Gumora and Arsenio, 2002; Djambazova-Popordanoska, 2016) in children. Although the past several decades have seen a steady increase in research investigating the consequences of maladaptive ER, factors that promote the development of adaptive ER remain poorly understood. However, there is evidence that ER is highly interrelated with executive functioning (EF; e.g., for a review, see Zelazo and Cunningham, 2007; Calkins and Marcovitch, 2010), and that individual differences in EF are predictive of ER abilities (e.g., Kieras et al., 2005; McDermott et al., 2009; for a review, see Schmeichel and Tang, 2015). As such, individual factors that enhance EF may be expected to promote the development of adaptive ER abilities.

Although seemingly distinct from ER, bilingualism, or proficiency in a second language (L2) is associated with advantages on a variety of EF tasks (see Adesope et al., 2010, for a meta-analysis; for a review, see Barac et al., 2014). Some EF tasks administered in research on bilingualism have also been used in ER research to assess cognitive control as it interacts with emotional processing (e.g., Bell and Wolfe, 2004; for a review, see Cole et al., 2004). Examining the interrelation between cognitive and emotional processing (Bell and Wolfe, 2004) may be key to explaining how proficiency in an L2 may also promote ER. The current study investigates whether bilingualism supports more adaptive ER strategies in school-aged children than is found for monolingual children.

Literature on ER has attracted significant attention due to its association with a variety of important developmental outcomes (for review, see Gross, 2002; Eisenberg et al., 2010). Although some controversy continues to exist over its constituents, Calkins and Hill (2007) view ER as the range of conscious and unconscious behaviors, skills, and strategies that change one’s emotional experience and expression either in an automatic or effortful way. Most definitions of ER also recognize interacting emotional and cognitive processes as integral to ER (for review, see Calkins and Marcovitch, 2010). These cognitive operations, including working memory, fall under the umbrella term of EF (Miyake et al., 2000; for a review, see Zelazo and Carlson, 2012). Working memory has been defined as a “cognitive system in which memory and attention interact to produce complex cognition” (Shipstead et al., 2015) and is a pivotal component of the EF system (Miyake et al., 2000). Working memory not only requires memory updating and retention but also relies on attentional control, which can vary between task conditions and challenge the EF system to different degrees (Miyake et al., 2000). Within ER contexts, EF interacts with emotional processing to modify appraisals, feelings, and behaviors in response to emotional experiences (for review, see Zelazo and Cunningham, 2007; Calkins and Marcovitch, 2010). Optimal ER development thus depends on the acquisition of cognitive skills such as working memory that allow the child to focus on task-relevant information with minimal interference from distracting and non-goal-oriented cues.

Research on emotion and working memory has primarily focused on adult clinical populations, but other research has investigated the emotion–cognition interaction in both clinical and non-clinical samples across development (Bradley et al., 1999; Bar-Haim et al., 2007; Ladouceur et al., 2009). One of the most popular paradigms used to assess working memory is the *n-*back task (for a review, see Owen et al., 2005; Meule, 2017). In this task, participants are asked to recall whether the location or identity of a target on the screen (e.g., the letter M) matches the location or identity of a stimulus presented *n* trials previously; memory load increases as *n* increases. Emotional faces (angry, sad, fearful, happy, neutral/calm) are often adopted as distracting emotional cues.

Studies investigating the impact of emotional valence on working memory report mixed findings. This is likely due to the extensive variability in the populations studied (typically vs. atypically developing, and different age groups), making it difficult to draw parallels between findings and particularly challenging to make predictions for typically developing children. Some authors find only significant slowing in response to negative emotional cues relative to positive or neutral ones (*healthy adult sample*: Kensinger and Corkin, 2003; *anxious sample of 8- to 30-year-olds*: Ladouceur et al., 2009), whereas others report no reaction time (RT) differences by emotion type and instead report impaired accuracy on trials with negative compared to neutral distractors (*adult controls and ADHD participants*: Marx et al., 2011). Others have reported varying speed-accuracy trade-offs by emotion type, including higher accuracy but slower RTs for negative compared to neutral stimuli in a non-verbal working memory task with a sample of schizophrenic participants (Becerril and Barch, 2011). This finding supports previous work showing that in emotionally dysregulated populations, aversive stimuli generate a significantly larger burden on the cognitive system than positive ones, depleting resources available for working memory (Bishop et al., 2004; Hare et al., 2005). For example, emotionally dysregulated, clinically depressed patients report an inability to disengage from pervasive negative thoughts (for a review, see Gotlib and Joormann, 2010), resulting in memory challenges as well as difficulty with planning and concentration (Paelecke-Habermann et al., 2005; Rose and Ebmeier, 2006).

Similar tendencies have been reported among clinically anxious and depressed children, showing differences in performance compared to healthy controls. Ladouceur et al. (2005) administered the Emotional *N*-back Task to a sample of 75 children (8–16 years of age) categorized into one of four groups: children who met criteria for an anxiety disorder, major depressive disorder, comorbid anxiety and depression, or were identified as a normal control group. In this version of the task, the distracting emotional stimuli were neutral, negative, and positive images in the background of the to-be-remembered letters. Their results showed that children with major depressive disorder and those with comorbid anxiety and depression had significantly longer RTs on the negative condition than on the neutral condition, whereas children in the normal control group had significantly longer RTs on the positive condition than on the neutral condition. Ladouceur et al. (2009) took a developmental approach to demonstrate ER changes with age within a sample of 8- to 30-year-old participants with low versus high levels of trait anxiety. The authors used the emotional face *N*-back task with 0-back and 2-back memory load conditions and three emotional face distractor types (neutral, fearful, and happy) as well as a control condition with shapes. Their findings revealed that individuals high in trait anxiety had slower RTs on the fearful 2-back memory-load condition than on the happy and neutral trials, but that the effect was greatest in younger participants. Conversely, individuals low in trait anxiety did not reveal any emotion effects, either in RT or accuracy rates. Taken together, these findings highlight that there are differences between how children and adults process distracting emotional information and that we continue to find inconsistent results when investigating interacting cognitive and emotional processing in typically developing children.

From a separate area of research, the cognitive benefit for bilingual individuals has been identified as enhanced performance on tasks requiring non-verbal EF (for a review, see Bialystok, 2017). Improvement in EF is believed to develop as a result of the well-documented coactivation of both languages within the bilingual brain, even when only one language is in use (e.g., Beauvillain and Grainger, 1987; Colomé, 2001; for a review, see Kroll et al., 2014). The practice of attending to one cue (one language) during interference from another “trains” the EF network (for a review, see Bialystok, 2015), becoming more effective throughout life and thereby extending the practice of verbal cognitive control to the non-verbal EF network (Green, 1998; Abutalebi et al., 2008; Luk et al., 2010). Evidence for the enhanced cognitive control in bilinguals comes from research using a variety of cognitive tasks with infants (Kovacs and Mehler, 2009), toddlers (Poulin-Dubois et al., 2011), young children (e.g., see Adesope et al., 2010, for a meta-analysis), and young adults (e.g., Costa et al., 2009). Recent findings have emerged that do not support these results with young adults (e.g., Paap and Greenberg, 2013) possibly due to differences in populations, criteria for bilingualism, or the nature of the experimental tasks used to assess cognitive ability (for a review, see Antoniou, 2019). However, the majority of the research points to bilingual benefits across a variety of cognitive control operations, especially where conflict conditions pose additional attentional demands on the EF system.

Given the central importance of working memory to EF, some bilingualism research has investigated differences in verbal and non-verbal working memory in children and adults, with mixed findings. Morales et al. (2013) conducted two studies that assessed working memory in 5-year-old (Study 1) and 5- to 7-year-old (Study 2) monolingual and bilingual children. In Study 1, the authors found that differences in performance between the groups emerged only on the most challenging condition of a Simon-type task, with bilingual children showing an EF advantage when a high level of conflict was present. In the second study, where children were required to recall the positions of frogs presented either simultaneously (easy) or sequentially (hard) within a 3 × 3 grid (Frog Matrices task), bilingual children had better accuracy on the more challenging sequential condition. Blom et al. (2014) investigated both visuospatial (Dot Matrix and Odd-One-Out tasks) and verbal working memory (Forward and Backward Digit Recall) performance in bilingual Turkish–Dutch children and Dutch monolingual controls from low socioeconomic backgrounds. Although no difference was found between the two language groups in 5-year-old children, by 6 years of age bilingual children showed overall benefits on the Dot Matrix task and the Backward Digit Recall task, both of which pose additional demand for EF over the other two tasks. While some have failed to reproduce this effect using simpler working memory measures (Engel de Abreu, 2011), bilingual children show advantages over their monolingual peers on conditions of heightened conflict.

In summary, several lines of evidence depict bilingual advantages in EF on tasks where successful performance depends on the ability to resolve conflict from competing cues and ignore interfering information or to maintain task rules in working memory. Ultimately, by monitoring language choice among competing linguistic systems, bilinguals must learn to more effectively regulate attention to distracting information, resulting in an EF system that is better equipped to support processes of working memory.

What are the implications of bilingualism for ER? Importantly, emotional and cognitive processes are highly interactive and integral to ER in that effective ER in emotional contexts depends on the EF system to process relevant information without being impaired by interfering emotional cues (Gray et al., 2002). Concurrently, literature on bilingualism shows evidence of strengthened cognitive control in dual-language users, resulting in greater selective attention to relevant information and reduced interference from distracting cues (Bialystok, 2015). It is thus reasonable to hypothesize that bilingualism may promote the development of more adaptive ER by strengthening the cognitive control system and all its constituents, including working memory. Furthermore, if bilingualism contributes to the development of self-regulatory abilities in emotionally challenging contexts at earlier stages of development than is found for monolinguals, then these enhanced abilities may also have implications for children’s psychosocial outcomes. However, research assessing ER differences between monolingual and bilingual children in this manner, and more comprehensively using standardized ER tasks, is largely lacking, and a direct evaluation of ER differences between these groups has not been undertaken.

The present study aimed to investigate the effect of bilingualism on cognitive and emotional processing that is integral to ER. The Emotional Face *N*-Back Task, an emotionally based EF task of working memory with three emotion conditions (angry, happy, and neutral), was used to examine differences in ER in school-aged monolingual and bilingual children. The overarching hypothesis was that bilingual children would demonstrate an overall advantage in working memory. Given that an EF advantage was expected in the bilingual group, and that the ability to modulate attention toward or away from emotionally salient information is a marker of ER and associated with EF, we anticipated finding evidence for ER benefits for bilinguals. Although the findings in this area with healthy children are mixed, it was predicted that the ER benefits for bilinguals would be most salient on the particularly challenging angry emotion trials, with the highest EF demands. This is the first study to compare these processes in healthy young children and evaluate the influence of bilingualism on ER. The novelty of this research will contribute to our understanding of differences in emotional processing between groups of children with different language experiences, over and beyond the known advantage of cognitive control in bilingual children.

## Materials and Methods

### Participants

One hundred and two children between 8- and 11-years old were recruited from four elementary schools. Based on caregiver reports of the child’s language background, an aggregate score was created to classify children as monolingual or bilingual. Nine children were removed from the study due to behavioral concerns that prevented them from completing the tasks. Complete data for analysis were available for 93 children, 48 monolinguals (*M* age = 9.3 years, *SD* = 0.6; 18 boys) and 45 bilinguals (*M* age = 9.4 years, *SD* = 0.5; 20 boys). The majority of children were born in Canada (78.5%), with 10 children born in the Philippines (10.8%) and the remainder being born in 10 different countries. Children in the bilingual group proficiently spoke a non-English language at home: Portuguese (*n* = 15), Philippine dialect (*n* = 12; Tagalog, Vasayan, or Ilonggo), Italian (*n* = 5), Spanish (*n* = 6), or seven other different languages (*n* = 7). School instruction was in English for all children.

### Procedure

Approval to test in the schools was obtained from the University Ethics Committee and from the school board’s ethics committee. The principal and teachers at each school agreed to have researchers introduce the study tasks to the children within their own classrooms. A packet of questionnaires was sent home with each child so that interested parents could complete the parental informed consent, the Language and Social Background Questionnaire (LSBQ), the Strengths and Weaknesses of Attention-Deficit/Hyperactivity Disorder Symptoms and Normal Behavior Scale (SWAN), and the Emotion Regulation Checklist (ERC). Before working with a child, qualified research assistants ensured that the complete packet had been returned to the teacher. Teachers were also asked to complete the ERC for each child that returned the packet of questionnaires.

**FIGURE 1:**
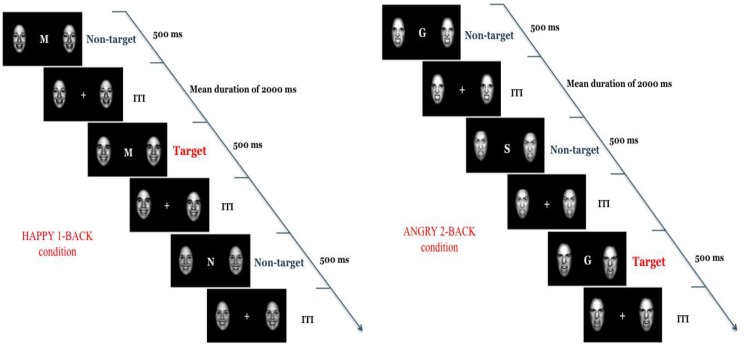
Emotional Face *N*-Back Task (adapted from Ladouceur et al., 2009). Children see one letter at a time presented on the screen and are asked to indicate whether the letter is the same as the letter they saw one screen back (1-back) or two screens back (2-back), creating two memory conditions. A face displaying an angry, happy, or neutral expression is shown on both sides of the letter.

Each child who returned a completed packet to their school was withdrawn from their classroom for approximately 45 min to complete the testing session. The procedure was explained to the child, and verbal assent was obtained prior to testing. During the session, each participant completed the Peabody Picture Vocabulary Test (PPVT), a standardized test of English proficiency, the Raven Standard Progressive Matrices (Raven), assessing spatial reasoning, and the Emotional Face *N*-Back Task (**Figure 1**), a task of ER. Upon completion of the *n*-back task, children were asked to subjectively rate the expression of a subset of angry, happy, and neutral faces that they had seen during the task to assess whether all children perceived the faces similarly. Throughout the session, children received stickers for completing each task. Research assistants made ongoing notes during the session to identify children whose behavior (talking, singing, refusal to continue, excessive fidgeting or movement, etc.) interfered with their ability to complete the tasks; these children were later removed from the study (*n* = 9). Each child was thanked for their participation and awarded a personalized certificate to recognize their effort before being walked back to their classroom.

### Questionnaires and Tasks

#### Language and Social Background Questionnaire (LSBQ; Anderson et al., 2018)

The LSBQ is completed by parents/guardians and contains questions pertaining to the child’s age, sex, handedness, time spent using video/computer games, and language fluency and use in different contexts. Parental education is indicated and used as a proxy for socioeconomic status (SES). SES was assessed as the average of mother’s and father’s education, using a 5-point scale with 1 indicating no high school diploma, 3 indicating some college or college diploma, and 5 indicating graduate or professional degree.

#### Peabody Picture Vocabulary Test (PPVT; Dunn and Dunn, 1997)

The PPVT is a standardized measure of receptive vocabulary. Children hear a word and are required to point to which one of four pictures corresponds with that word. Testing proceeds until the child makes eight errors within a block of 12 words. The PPVT normally takes 15–20 min to complete. Scores are standardized based on the participant’s age (μ = 100, *SD* = 15). The PPVT has a high reliability (>0.90) across a variety of measures (i.e., internal consistency, split-half, test–retest) and a 0.91 correlation with the Wechsler’s Intelligence Scale for Children’s measure of verbal ability (WISC-III; Wechsler, 1991).

#### Raven Standard Progressive Matrices (Raven Test; Raven et al., 1996)

The Raven test is a standardized test of non-verbal spatial reasoning. Children view test figures and chose which item from a set of six options provides the best completion. The task normally takes 10–15 min to complete with children. Results are converted to standardized scores based on the participant’s age (μ = 100, *SD* = 15). The predictive validity of the Raven test is around 0.70, whereas test–retest reliability and internal consistency coefficients range between 0.80 and 0.93 (Raven et al., 1996).

#### Strengths and Weaknesses of Attention-Deficit/Hyperactivity Disorder Symptoms and Normal Behavior Scale (SWAN; Swanson et al., 2001)

The SWAN questionnaire is completed by a child’s parent or guardian and teacher. The SWAN includes 18 items that are associated with the characteristic symptoms assessed for a diagnosis of ADHD as described in the DSM-5 (American Psychiatric Association, 2013). These include nine symptoms related to *inattention* (e.g., “Stays focused on tasks and activities”), six symptoms related to *hyperactivity* (e.g., “Can sit without constant fidgeting or squirming”), and three items related to *impulsivity* (e.g., “Easily waits turn, such as standing in line-ups”). Each item is positively worded and was modified slightly from the original test to improve ease of reading and decrease word difficulty for parents/guardians who may struggle with understanding English (e.g., “Sustains attention on tasks or play activities” was changed to “Stays focused on tasks and activities”). A guardian and teacher rated the child on each item using a 4-point Likert scale ranging from “Far below average” (1) to “Far above average” (4). Higher scores are indicative of better attentional abilities, lower hyperactivity, and lower impulsivity. The SWAN has excellent internal consistency and reliability (Young et al., 2009; Lakes et al., 2012).

#### Emotion Regulation Checklist (ERC; Shields and Cicchetti, 1997)

The ERC is a 24-item measure intended to assess the frequency of children’s displays of affective behaviors. Parents/caregivers and teachers rate the frequency of the behavior using a 4-point scale. The raw scores generate two subscales: (1) ER, which assesses socially appropriate emotional responses and empathy, and (2) lability-negativity, which assess arousal more broadly, focusing on anger, dysregulation, and mood lability. High internal consistency has been shown for both the lability-negativity and ER subscales, with Cronbach’s alphas of 0.96 and 0.83, respectively (Shields and Cicchetti, 1997).

#### Emotional Face *N*-Back Task (*N*-back; Adapted From Ladouceur et al., 2009)

The emotional variant of the *n*-back paradigm is designed to examine the interference effect of emotional information on working memory performance. The task consisted of two memory conditions (1-back and 2-back), with blocked emotional (angry, happy) and neutral conditions, for each level of difficulty. Letters were presented in the middle of the screen and two of the same emotional faces were presented simultaneously on both sides of the letter to act as the emotional distractors (see **Figure 1**). In the 1-back condition, participants were asked if the letter was the same as the letter on the previous trial (target, “yes”) or not (non-target, “no”). In the 2-back condition, participants decided whether the current letter matched the trial that was presented *two* trials previously (target) or not (non-target). Responses were made using two mice, one assigned to each response, with the dominant hand assigned to target trials and the non-dominant hand to non-target trials. Angry, happy, and neutral faces were taken from the NimStim set available at www.macbrain.org (Tottenham et al., 2009), and modified so that only an oval-shaped face was visible, without hair or a neck. Each emotion block was made up of 15 target (“yes”) and 25 non-target (“no”) trials. The task took approximately 15 min to complete.

#### Affect Rating

After completing the *n*-back task children were presented with the angry, happy, and neutral expressions of three NimStim actors whose faces they had seen during the task. The NimStim actors were two females and one male, all demographically diverse. Children chose one adjective to describe the expression on each face without being told whether the face was meant to portray a happy, angry, or neutral expression. The purpose was to assess whether there were differences in how monolingual and bilingual children perceive emotional expressions. The top three descriptive words used to identify each emotional face were compared between the two language groups. The findings were also used to determine whether the child descriptions found in the current study replicated previous findings from the child literature depicting neutral faces as more aversive to children than happy faces.

## Results

The background measures for age, SES (parental education), vocabulary knowledge (PPVT), and nonverbal cognitive functioning (Raven test), are reported in **Table 1**. One-way ANOVAs for language group showed no differences between children in the two groups on any of these measures (all *p*s > 0.14). Mean scores on the subscales of the SWAN (attention, hyperactivity, impulsivity) and the ERC (ER, negativity/lability) are reported in **Table 2** for teacher and parent/guardian reports. One-way ANOVAs for language group showed no differences between the teacher ratings for children in the two groups on any of the subscales (all *p*s > 0.31), but parent ratings revealed that monolingual and bilingual children were rated similarly on hyperactivity (*p* = 0.76), impulsivity (*p* = 0.99), ER (*p* = 0.63), and negativity/lability (*p* = 0.41), but differently on attention, *F* (1,90) = 4.53, *p* = 0.04, $\eta_{p}^{2}$ = 0.05, with bilingual children (3.1) being rated as more attentive than monolingual children (2.8).

**Table 1:** Mean score, standard deviation, and range for background measures by language group.

	Monolingual (*n* = 48)		Bilingual (*n* = 45)	
		
Background measure	*M* (SD)	Range	*M* (SD)	Range
Age in months	9.3 (0.6)	8.3–10.5	9.4 (0.5)	8.3–10.3
SES^∗^	3.4 (1.0)	2–5	3.1 (1.3)	1–5
PPVT	101.6 (13.5)	81–135	97.5 (13.2)	67–125
Raven test	101.3 (14.9)	75–130	97.8 (12.9)	75–120


^∗^SES (socioeconomic status) was measured as the average of maternal and paternal education level (3 = completed college).

**Table 2:** Mean score and standard deviation for reports made by teachers and parents on children’s behavior by language group.

	Monolingual (*n* = 48)		Bilingual (*n* = 45)	
		
Teacher and parental reports on child behavior	Teacher *M* (SD)	Parent *M* (SD)	Teacher *M* (SD)	Parental *M* (SD)
**SWAN (out of 4)**				
Attention	2.8 (0.8)	2.8 (0.7)	3.0 (0.8)	3.1 (0.6)^∗^
Hyperactivity	3.1 (0.9)	3.0 (0.7)	3.2 (0.7)	3.1 (0.8)
Impulsivity	3.2 (0.9)	3.0 (0.7)	3.2 (0.8)	3.0 (0.8)

**ERC (out of 4)**				
Emotion regulation	3.2 (0.6)	3.3 (0.4)	3.2 (0.6)	3.4 (0.4)
Negativity/lability	3.4 (0.6)	3.2 (0.4)	3.5 (0.5)	3.3 (0.4)


^∗^Significant difference in ratings between language groups, *p* < 0.05.

**FIGURE 2:**
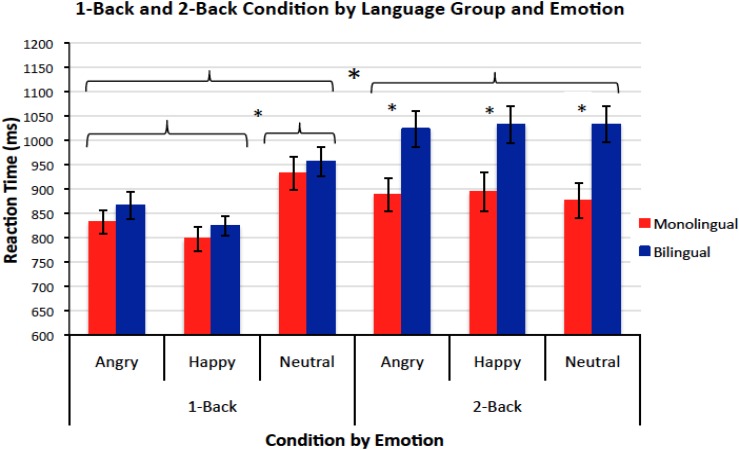
Mean reaction times (and standard errors) on the Emotional Face *N*-back Task by condition (1-back vs. 2-back), emotion (angry, happy, neutral), and language group (monolingual vs. bilingual).

The outcomes for accuracy and RT on the Emotional Face *N*-back task are reported in **Table 3** and **Figure 2**, respectively. Accuracy on correct target trials was analyzed using a three-way ANOVA for *n*-back condition (1-back, 2-back), emotion (angry, happy, neutral), and language group (monolingual, bilingual). The analysis revealed a main effect of condition, *F* (1,91) = 239.97, *p* < 0.001, $\eta_{p}^{2}$ = 0.73, with children scoring higher on the 1-back (75.78%) than on the more challenging 2-back (52.70%) condition, and a main effect of language group, *F* (1,91) = 9.71, *p* < 0.01, $\eta_{p}^{2}$ = 0.08, with bilingual children (67.83%) outperforming monolingual children (60.6%). There was no main effect of emotion, *p* = 0.26, and no interactions, all *F*s*<* 1.37, *p*s > 0.26.

**Table 3:** Mean score and standard deviation for accuracy on the Emotional Face *N*-back Task by language group.

	Monolingual (*n* = 48)	Bilingual (*n* = 45)
		
Working memory by emotion condition	*M* (SD)	*M* (SD)
**Accuracy on target (“yes”) trials (% correct)**		
1-back		
Angry	71.4 (15.3)	78.67 (12.5)^∗^
Happy	73.9 (13.1)	81.6 (12.3)^∗^
Neutral	71.5 (17.9)	77.6 (14.4)^∗^
2-back		
Angry	49.4 (17.6)	54.5 (21.5)^∗^
Happy	49.0 (17.5)	57.0 (17.6)^∗^
Neutral	48.8 (17.8)	57.5 (14.9)^∗^

**Accuracy on nontarget (“no”) trials (% correct)**		
1-back		
Angry	86.8 (16.1)	90.2 (12.3)
Happy	86.5 (17.0)	90.0 (12.1)
Neutral	85.5 (15.0)	86.1 (14.7)
2-back		
Angry	80.5 (17.3)	78.0 (18.8)
Happy	78.8 (16.5)	80.0 (13.8)
Neutral	78.3 (18.4)	78.6 (15.4)


^∗^Significant difference in ratings between language groups, *p* < .05.

Accuracy on *nontarget* trials was also investigated to determine whether the higher accuracy scores on target trials for the bilingual group reflected a response bias to say “yes” (i.e., identify more trials as *target* trials). Nontarget trials were analyzed using a three-way ANOVA for condition, emotion, and language group. The results revealed only a main effect of *n-*back condition, *F* (1,91) = 27.81, *p* < 0.001, $\eta_{p}^{2}$ = 0.22, with children correctly identifying more nontarget trials (“no” response) on the 1-back (87.48%) than on the 2-back (79.04%), as expected. There was no main effect of language group (*p* = 0.64) or emotion (*p* = 0.16), or any interactions, all *F*s < 0.60, *p*s > 0.35.

Reaction time data for the Emotional Face *N*-back task were analyzed the same way as accuracy data, using a three-way ANOVA for *n-*back condition, emotion, and language group on target trials (see **Figure 2**). There was a main effect for condition, *F* (1,91) = 21.86, *p* < 0.001, $\eta_{p}^{2}$ = 0.18, with children performing slower on the more challenging 2-back (958 ms) than the 1-back (868 ms). A main effect of emotion, *F* (1,186) = 13.03, *p* < 0.001, $\eta_{p}^{2}$ = 0.13, revealed that RTs were significantly slower on the neutral trials (949 ms) than on angry (902 ms), *p* < 0.001, or happy (886), *p* < 0.001, trials, with no difference between the latter two, *p* = 0.57. Furthermore, a two-way interaction of *n-*back condition and emotion, *F* (2,182) = 16.71, *p* < 0.001, $\eta_{p}^{2}$ = 0.08, revealed that the effect of emotion was present on the 1-back condition, *F* (2,182) = 39.09, *p* < 0.001, $\eta_{p}^{2}$ = 0.10, but not on the 2-back condition, *p* = 0.89. Finally, a main effect of language group, *F* (1,91) = 5.46, *p* = 0.02, $\eta_{p}^{2}$ = 0.07, revealed that bilingual children (956 ms) were significantly slower than their monolingual peers (870 ms), but a two-way interaction of condition and language group, *F* (1,91) = 9.03, *p* < 0.01, $\eta_{p}^{2}$ = 0.08, restricted this difference to the 2-back condition *F* (1,91) = 9.10, *p* < 0.01, $\eta_{p}^{2}$ = 0.10, with no difference between groups in the 1-back condition, *p* = 0.23.

A correlation was computed between accuracy and RT for each condition to determine whether there were speed-accuracy trade-offs. There were no significant correlations in the 1-back condition, *r* (93) = 0.19, *p* = 0.16, but the relation was significant in the 2-back condition, *r* (93) = 0.34, *p* = 0.001. Given that bilingual children performed significantly slower than monolingual children on the 2-back, a correlation was run separately by language group to determine whether the speed-accuracy trade-off in the 2-back was driven by the bilingual group. The correlation revealed that the speed-accuracy trade-off was significant for the bilingual children, *r* (45) = 0.34, *p* = 0.02, but only marginal for monolingual children, *r* (48) = 0.24, *p* = 0.09. However, the Fisher r-to-z transformation revealed that the difference between the two correlations was not significant, *p* = 0.61.

Reaction time on *nontarget* trials was also investigated using a three-way ANOVA for *n-*back condition, emotion, and language group. There was a main effect of emotion, *F* (2,182) = 19.29, *p* < 0.001, $\eta_{p}^{2}$ = 0.18, with children performing more slowly on neutral trials (969 ms) than on angry (909 ms) or happy (901 ms) trials, all *p*s < 0.001, with no difference in speed of performance between angry and happy emotions, *p* = 1.00. A main effect of language group was also found, *F* (1,91) = 5.30, *p* = 0.02, $\eta_{p}^{2}$ = 0.07, in which bilingual children (971 ms) were slower than their monolingual peers (880 ms). An interaction of *n-*back condition by emotion, *F* (2,182) = 21.06, *p* < 0.001, $\eta_{p}^{2}$ = 0.20, revealed that differences in speed of responding between emotion blocks emerged only on the 1-back version of the task, *F* (2,182) = 43.13, *p* < 0.001, $\eta_{p}^{2}$ = 0.42, where children performed slower on angry (900 ms) than happy (856 ms) trials, slower on neutral (995 ms) than angry trials, and slower on happy than neutral trials, all *p*s < 0.01; no differences between angry (918 ms), happy (946 ms), and neutral (944 ms) trials were found on the 2-back version of the task, all *p*s > 0.14. No other main or interaction effects were found, all *F*s < 2.5, *p*s > 0.14.

Children’s affect ratings of nine preselected facial expressions (three per emotional type) used in the task were recorded and evaluated. The three most frequently occurring words used by children in each language group were tabulated by facial expression and emotion (**Table 4**). These top three words were then inspected to determine whether there were notable differences in the valence of the words within each emotion block and across language groups. The word inspection revealed that children used all positively valenced words (e.g., happy, excited, joyful) to describe the three standardized happy faces, and they rated all standardized angry faces using negatively valenced words (e.g., angry, mad, scary, furious), as expected. The three standardized neutral faces rated by the children generated the greatest amount of variety in valence. Children described neutral faces using words with a positive (i.e., happy), negative (i.e., shocked, sad, scared, serious), and neutral (i.e., normal, bored, no emotion/expression) valence. No outstanding differences were detected between language groups in the words children selected to describe any of the actors’ faces in either emotion block.

**Table 4:** Three most commonly used words by children to describe the emotional expressions of three actors with standardized angry, happy, and neutral affects viewed during the Emotional Face *N*-back Task by language group.

Standardized *n*-back faces by emotion	Actor 1 (Asian, female)		Actor 2 (African American, female)		Actor 3 (Caucasian, male)	
			
	Monolingual (*n* = 48)	Bilingual (*n* = 45)	Monolingual (*n* = 48)	Bilingual (*n* = 45)	Monolingual (*n* = 48)	Bilingual (*n* = 45)
Angry	Angry (34)	Angry (36)	Angry (31)	Angry (30)	Angry (22)	Angry (18)
	Mad (14)	Mad (9)	Mad (13)	Mad (11)	Mad (17)	Mad (22)
	–	Scary (1)	Furious (2)	Frustrated (1)	Frustrated (2)	Furious (2)
				Furious (1)		
Happy	Happy (48)	Happy (46)	Happy (35)	Happy (36)	Happy (46)	Happy (40)
	–	–	Excited (6)	Excited (10)	Glad (1)	Excited (2)
			Energized (1)	–	Joyful (1)	Silly (2)
			Joyful (1)			Cheerful (2)
Neutral	Surprised (19)	Surprised (16)	Sad (11)	Sad (19)	Normal (14)	Normal (14)
	Shocked (5)	Shocked (6)	Bored (9)	Bored (6)	Bored (5)	Happy (7)
	Sad (4)	Scared (5)	Normal (7)	Normal (5)	No emotion/expression (4)	Serious (4)


## Discussion

The present study investigated the interrelation between cognitive and emotional processing in typically developing monolingual and bilingual children. Children in the two language groups were similar on age, SES, English proficiency, and nonverbal cognitive functioning. Parents provided information on children’s language background, and both parents and teachers reported on children’s emotional and behavioral functioning. All children were tested using the Emotional Face *N*-Back Task, an ER task assessing working memory within an emotional context.

Ratings of attention obtained from the SWAN showed that all children were rated similarly by teachers, but bilingual children were rated by parents as significantly more attentive than monolingual children. Although it is common to find discrepancies between informants when gathering ratings on children’s behavior (Gresham et al., 2010; Wray et al., 2013), little is known about characteristics that predict discrepancies in ratings. Nonetheless, parental ratings indicating greater attention in bilingual children bring to light an important consideration, namely the frame of reference of the informant. Teachers have experience with children from different communities and cultures and so have a wide frame of reference for rating children’s performance relative to their same-aged peers. Parents, in contrast, may be limited to observing the children living in their own community or even household. Furthermore, parents of monolingual or bilingual children may have different culture-specific expectations that influence their parenting practices and expectations for normative development. Thus, while it was beyond the scope of this study, future bilingualism researchers may consider gathering information on familial expectations and background as these may be related to differences between language groups in parental ratings of children’s behavior.

The Emotional Face *N*-back Task was used to investigate differences in ER between monolingual and bilingual children by manipulating working memory load (1-back or 2-back) and emotional distraction (angry, happy, and neutral faces). As expected, all children were more accurate and faster on the easier condition than on the more challenging condition of the task. Also in line with our predictions, bilingual children demonstrated better cognitive performance on both working memory conditions. Specifically, depending on the emotion block and condition, accuracy rates of bilingual children ranged from 6% to 9% higher than those of their monolingual peers. This observed working memory advantage for bilingual children supports research highlighting the cognitive benefits of bilingualism on a variety of EF tasks (Adesope et al., 2010).

The findings also revealed significantly slower RTs on neutral emotion trials than on angry and happy trials for children in both language groups, but only on the 1-back condition. Thus, the easy working memory condition showed differences between the emotional stimuli but the difficult working memory condition did not, presumably because the effort required for the working memory response overwhelmed the more subtle difference between emotion conditions. As in previous research with emotional *n*-back tasks (e.g., Ladouceur et al., 2005; Cromheeke and Mueller, 2016; Villemonteix et al., 2017), accuracy of responses was not impacted by the emotion condition.

The longer RTs on neutral trials may reflect the challenges children experienced in accurately labeling neutral faces. When asked to generate affect ratings for the angry, happy, and neutral emotional expressions for the subset of stimuli used in the task, all children gave accurate positively valenced ratings to happy faces and negatively valenced ratings to angry faces, but neutral faces elicited variable responses, ranging from positively to negatively valenced descriptions. This difficulty in interpreting neutral faces has been found on previous emotion recognition tasks (Kujawa et al., 2014; see Herba et al., 2006; Thomas et al., 2007, for research on age-related changes in emotion recognition). Consequently, the slow responses to neutral emotion trials may reflect children’s difficulty in interpreting ambiguous neutral face stimuli.

The absence of an emotion effect on the 2-back condition may have been influenced by the difficulty that this task posed for children this age in both language groups. Support for this interpretation comes from the neuroscientific literature; studies using neuroimaging during ER tasks have found that increasing requirements for EF can override emotional effects (Hart et al., 2010; Mueller et al., 2017). For example, Erk et al. (2006) found no effects of emotional cueing on working memory accuracy on their ER task; however, the fMRI results revealed a valence-specific regulation effect on brain regions whereby participants had significantly reduced activity in brain areas responsible for emotional processing during high cognitive effort conditions than during low cognitive effort conditions, and significantly greater recruitment of regions implicated in working memory as the complexity of the task increased. This research supports our behavioral findings in that the effects of emotional context tend to be reduced under conditions of high cognitive effort as participants attempt to meet the demands of increasing task complexity (e.g., on the 2-back version of the task).

In this context, it might therefore be expected that bilingual children would respond differently than monolingual children in the 1-back version of the task, but this did not happen. Overall, the study failed to capture the anticipated differences in ER between the language groups. Instead, the main finding was that bilingual children were significantly slower than their monolingual peers on the 2-back condition of the Emotional Face *N*-back Task.

In healthy adult populations, researchers find that positive emotional stimuli are generally processed more quickly and automatically than emotionally neutral stimuli (see Pool et al., 2016, for a meta-analysis), as was found in the present study. Research with typically developing children is sparse and has generated mixed results; however, it is generally accepted that children’s ability to modulate attention toward or away from emotionally salient information is a marker of ER that distinguishes healthy from at-risk or atypically developing children (e.g., Shackman et al., 2007; Nuske et al., 2017), and that responses are less consistent than those observed in adults. For example, Mueller et al. (2012) tested healthy versus anxious 12-year-old youth using an antisaccade task with emotional faces and found that healthy youth were more accurate during angry trials and happy trials relative to neutral trials, but revealed no emotion effects in RT. However, typically developing children have also been shown to demonstrate longer RTs on positive emotional conditions than on neutral emotional conditions (Ladouceur et al., 2005). Conversely, studies with anxious children, youth, and adults on ER tasks consistently find a threat bias, also described as biased allocation of attentional resources toward threatening stimuli, which is reflected in longer RTs on trials with aversive stimuli (Ladouceur et al., 2005, 2009; for a review, see Williams et al., 1997). Similar findings have been observed in depressed individuals, whose responses are characterized by impaired disengagement from negative stimuli and deficits in cognitive control while processing negative information (for a review, see Gotlib and Joormann, 2010). Taken together, prolonged engagement with emotionally threatening information is believed to be mediated by deficits in cognitive control. However, our finding that bilingual children exhibited longer RTs, particularly on the 2-back, cannot be explained by this theoretical proposal, because: (1) the slowing on (or lack of quick disengagement from) emotional stimuli for bilinguals was generalized to the whole 2-back condition and was nonspecific to either emotion condition, and (2) bilingual children demonstrated overall enhanced working memory relative to monolingual children, with no cognitive deficits noted across any conditions on the task. As such, it is reasonable to assume that there is a continuum between typical capture of attention by emotional cues and dysregulated or maladaptive emotional processing.

A possible explanation for the longer RTs for bilinguals and lack of ER differences between the language groups may lie in differences in monitoring and cognitive flexibility (or shifting) abilities between the groups (e.g., Bialystok and Viswanathan, 2009; Prior and MacWhinney, 2010). Shifting or cognitive flexibility is a component of EF, and the ability to think flexibly that includes switching strategies or responses as task demands change (Miyake et al., 2000). Vitiello et al. (2011) have linked enhanced cognitive flexibility to school success in children. It is notable that children in both language groups accommodated the difficulty of the 2-back condition by slowing down, but only the bilinguals maintained high accuracy in the difficult condition. Hur et al. (2017) observed that particularly on a more challenging task as the 2-back, “participants’ efforts are generally focused more on performing the task accurately than responding as fast as they can” (p. 4). Many studies show an increase in RT and decrease in accuracy with increasing task difficulty on *n*-back tasks (for a review, see Meule, 2017). Therefore, the slowing for bilingual children cannot be explained by impaired cognitive processing within emotional contexts, but may reflect normal development in healthy bilingual children who are better able than monolingual children to adjust their behavior to task demands.

In summary, this study demonstrated advantages in working memory for bilingual children compared to monolingual children, consistent with previous research showing EF benefits in bilingual individuals, but no evidence for better ER in bilinguals. ER has not been previously investigated with monolingual and bilingual individuals using a working memory task outside of the linguistic context. Although behavioral responses to negative emotional stimuli have commonly been studied within the context of dysregulation and maladjustment, and responses to positive emotions have been studied within the context of healthy socioemotional development, viewing child behavior through this narrow lens may be an oversimplification of functioning and undermine the importance of individual differences that modulate interacting cognitive and emotional processing. Continued research using ER tasks such as the Emotional Face *N*-back Task has the potential of advancing our understanding of the developmental mechanisms underlying ER in children, and more specifically in elucidating any differences in emotional processing between children with different language experiences.

## Ethics Statement

This research was approved by the Human Participants Review Committee of York University. Parents signed informed consent prior to the experiment and children provided verbal assent before each task.

## Author Contributions

MJ conducted the study under the supervision of EB in partial fulfillment of requirements for the degree of Doctor of Philosophy.

## Conflict of Interest Statement

The authors declare that the research was conducted in the absence of any commercial or financial relationships that could be construed as a potential conflict of interest. The reviewer LF-P and handling Editor declared their shared affiliation at the time of the review.

## References

[B1] Abutalebi J. Annoni J. M. Zimine I. Pegna A. J. Seghier M. L. Lee-Jahnke H. (2008). Language control and lexical competition in bilinguals: an event-related FMRI study. *Cereb. Cortex* 18 1496–1505. 10.1093/cercor/bhm182 17947346

[B2] Adesope O. Lavin T. Thompson T. Ungerleider C. (2010). A systematic review and Meta-analysis of the cognitive correlates of bilingualism. *Rev. Educ. Res.* 80 207–245. 10.3102/0034654310368803

[B3] American Psychiatric Association (2013). *Diagnostic and Statistical Manual of Mental Disorders*, 5th Edn. Washington, DC: Author 10.1176/appi.books.9780890425596

[B4] Anderson J. A. E. Mak L. Chahi A. K. Bialystok E. (2018). The language and social background questionnaire: assessing degree of bilingualism in a diverse population. *Behav. Res. Methods* 1–14 250–263. 10.3758/s13428-017-0867-9 28281208 PMC5591752

[B5] Antoniou M. (2019). The advantages of bilingualism debate. *Annu. Rev. Linguist.* 5 1–21. 10.1146/annurev-linguistics011718-011820

[B6] Barac R. Bialystok E. Castro D. C. Sanchez M. (2014). The cognitive development of young dual language learners: a critical review. *Early Childhood Res. Q.* 29 699–714. 10.1016/j.ecresq.2014.02.003 25284958 PMC4180217

[B7] Bar-Haim Y. Lamy D. Pergamin L. Bakermans-Kranenburgh M. J. van Ijzendoorn M. H. (2007). Threat-related attentional bias in anxious and nonanxious individuals: a meta-analytic study. *Psychol. Bull.* 133 1–24. 10.1037/0033-2909.133.1.1 17201568

[B8] Beauvillain C. Grainger J. (1987). Accessing interlexical homographs: some limitations of a language-selective access. *J. Mem. Lang.* 26 658–672. 10.1016/0749-596X(87)90108-2

[B9] Becerril K. Barch D. (2011). Influence of emotional processing on working memory in schizophrenia. *Schizophrenia Bull.* 37 1027–1038. 10.1093/schbul/sbq009 20176860 PMC3160211

[B10] Bell M. A. Wolfe C. D. (2004). Emotion and cognition: an intricately bound developmental process. *Child Dev.* 75 366–370. 10.1111/j.1467-8624.2004.00679.x 15056192

[B11] Bialystok E. (2015). Bilingualism and the development of executive function: the role of attention. *Child Dev. Perspect.* 9 117–121. 10.1111/cdep.12116 26019718 PMC4442091

[B12] Bialystok E. (2017). The bilingual adaptation: how minds accommodate experience. *Psychol. Bull.* 143 233–262. 10.1037/bul0000099 28230411 PMC5324728

[B13] Bialystok E. Viswanathan M. (2009). Components of executive control with advantages for bilingual children in two cultures. *Cognition* 112 494–500. 10.1016/j.cognition.2009.06.014 19615674 PMC2755257

[B14] Bishop S. Duncan J. Brett M. Lawrence A. D. (2004). Prefrontal cortical function and anxiety: controlling attention to threat-related stimuli. *Nat. Neurosci.* 7 184–188. 10.1038/nn1173 14703573

[B15] Blom E. Kuntay A. C. Messer M. Verhagen J. Leseman P. (2014). The benefits of being bilingual: working memory in bilingual turkish-dutch children. *J. Exp. Child Psychol.* 128 105–119. 10.1016/j.jecp.2014.06.007 25160938

[B16] Bradley B. P. Mogg K. White J. Groom C. de Bono J. (1999). Attentional bias for emotional faces in generalized anxiety disorder. *Br. J. Clin. Psychol.* 38 267–278. 10.1348/014466599162845 10532148

[B17] Calkins S. D. Hill A. (2007). “Caregiver influences on emerging emotion regulation: Biological and environmental transactions in early development,” in *Handbook of Emotion Regulation*, ed. Gross J. (New York, NY: Guilford Press), 229–248.

[B18] Calkins S. D. Marcovitch S. (2010). “Emotion regulation and executive functioning in early development: Integrated mechanisms of control supporting adaptive functioning,” in *Child Development: At the Intersection of Emotion and Cognition*, eds Calkins S. D. Bell M. A. (Washington, DC: APA Press), 37–58.

[B19] Cole P. M. Deater-Deckert K. (2009). Emotion regulation, risk, and psychopathology. *J. Child Psychol. Psychiatry* 50 1327–1330. 10.1111/j.1469-7610.2009.02180.x 19843190

[B20] Cole P. M. Martin S. Dennis T. (2004). Emotion regulation as a scientific construct: methodological challenges and directions for child development research. *Child Dev.* 75 317–333. 10.1111/j.1467-8624.2004.00673.x 15056186

[B21] Colomé À (2001). Lexical activation in bilinguals’ speech production: language-specific or language-independent? *J. Mem. Lang.* 45 721–736. 10.1006/jmla.2001.2793

[B22] Costa A. Hernández M. Costa-Faidella J. Sebastián-Gallés N. (2009). On the bilingual advantage in conflict processing: now you see it, now you don’t. *Cognition* 113 135–149. 10.1016/j.cognition.2009.08.001 19729156

[B23] Cromheeke S. Mueller S. C. (2016). The power of a smile: stronger working memory effects for happy faces in adolescents compared to adults. *Cogn. Emot.* 30 288–301. 10.1080/02699931.2014.997196 25650124

[B24] Djambazova-Popordanoska S. (2016). Implications of emotion regulation on young children’s emotional wellbeing and educational achievement. *Educ. Rev.* 68 497–515. 10.1080/00131911.2016.1144559

[B25] Dunn L. M. Dunn L. M. (1997). *Peabody Picture Vocabulary Test-III.* Circle Pines, MN: American Guidance Service.

[B26] Eisenberg N. Spinrad T. L. Eggum N. D. (2010). Emotion-related self-regulation and its relation to children’s maladjustment. *Annu. Rev. Clin. Psychol.* 6 495–525. 10.1146/annurev.clinpsy.121208.131208 20192797 PMC3018741

[B27] Engel de Abreu P. M. (2011). Working memory in multilingual children: is there a bilingual effect? *Memory* 19 529–537. 10.1080/09658211.2011.590504 21864216

[B28] Erk S. Abler B. Walter H. (2006). Cognitive modulation of emotion anticipation. *Eur. J. Neurosci.* 24 1227–1236. 10.1111/j.1460-9568.2006.04976.x 16930447

[B29] Gotlib I. H. Joormann J. (2010). Cognition and depression: current status and future directions. *Annu. Rev. Clin. Psychol.* 6 285–312. 10.1146/annurev.clinpsy.121208.131305 20192795 PMC2845726

[B30] Gray J. R. Braver T. S. Raichle M. E. (2002). Integration of emotion and cognition in the lateral prefrontal cortex. *Proc. Natl. Acad. Sci. U.S.A.* 99 4115–4120. 10.1073/pnas.062381899 11904454 PMC122657

[B31] Green D. W. (1998). Mental control of the bilingual lexico-semantic system. *Bilingualism* 1 67–81. 10.1017/S1366728998000133 29368141

[B32] Gresham F. M. Elliott S. N. Cook C. R. Vance M. J. Kettler R. (2010). Cross-informant agreement for social skill and problem behavior ratings: an investigation of the Social Skills Improvement System – Rating Scales. *Psychol. Assess.* 22 157–166. 10.1037/a0018124 20230162

[B33] Gross J. J. (2002). Emotion regulation: affective, cognitive, and social consequences. *Psychophysiology* 39 281–291. 10.1017/S0048577201393198 12212647

[B34] Gumora G. Arsenio W. F. (2002). Emotionality, emotion regulation, and school performance in middle school children. *J. Sch. Psychol.* 40 395–413. 10.1016/S0022-4405(02)00108-5

[B35] Hare T. A. Tottenham N. Davidson M. C. Glover G. H. Casey B. J. (2005). Contributions of amygdala and striatal activity in emotion regulation. *Biol. Psychiatry* 57 624–632. 10.1016/j.biopsych.2004.12.038 15780849

[B36] Hart S. J. Green S. R. Casp M. Belger A. (2010). Emotional priming effects during Stroop task performance. *Neuroimage* 49 2662–2670. 10.1016/j.neuroimage.2009.10.076 19883772 PMC2818423

[B37] Hawkins J. Catalano R. Kosterman R. Abbott R. Hill K. (1999). Preventing adolescent health-risk behaviors by strengthening protection during childhood. *Arch. Pediatr. Adolesc. Med.* 153 226–234. 10.1001/archpedi.153.3.226 10086398

[B38] Herba C. M. Landau S. Russell T. Ecker C. Phillips M. L. (2006). The development of emotion processing in children: effects of age, emotion, and intensity. *J. Child Psychol. Psychiatry* 47 1098–1106. 10.1111/j.1469-7610.2006.01652.x 17076748

[B39] Holley S. R. Ewing S. T. Stiver J. T. Bloch L. (2017). The relationship between emotion regulation, executive functioning, and aggressive behaviors. *J. Interpers. Violence* 32 1692–1707. 10.1177/0886260515592619 26130684

[B40] Hur J. Iordan A. D. Dolcos F. Berenbaum H. (2017). Emotional influences on perception and working memory. *Cogn. Emot.* 31 1294–1302. 10.1080/02699931.2016.1213703 27685764

[B41] Kensinger E. A. Corkin S. (2003). Effect of negative emotional content on working memory and long-term memory. *Emotion* 3 378–393. 10.1037/1528-3542.3.4.378 14674830

[B42] Kieras J. E. Tobin R. M. Graziano W. G. Rothbart M. K. (2005). You can’t always get what you want: effortful control and children’s responses to undesirable gifts. *Psychol. Sci.* 16 391–396. 10.1111/j.0956-7976.2005.01546.x 15869699

[B43] Kovacs A. M. Mehler J. (2009). Cognitive gains in 7-month-old bilingual infants. *Proc. Natl. Acad. Sci. U.S.A.* 106 6556–6560. 10.1073/pnas.0811323106 19365071 PMC2672482

[B44] Kroll J. F. Bobb S. C. Hoshino N. (2014). Two languages in mind: bilingualism as a tool to investigate language, cognition, and the brain. *Curr. Dir. Psychol. Sci.* 23 159–163. 10.1177/0963721414528511 25309055 PMC4191972

[B45] Kujawa A. Dougherty L. Durbin C. E. Laptook R. Torpey D. Klein D. N. (2014). Emotion recognition in preschool children: associations with maternal depression and early parenting. *Dev. Psychopathol.* 26 159–170. 10.1017/S0954579413000928 24444174 PMC3898589

[B46] Ladouceur C. D. Dahl R. E. Williamson D. E. Birmaher B. Ryan N. D. Casey B. J. (2005). Altered emotional processing in pediatric anxiety, depression, and comorbid anxiety-depression. *J. Abnorm. Child Psychol.* 33 165–177. 10.1007/s10802-005-1825-z 15839495

[B47] Ladouceur C. D. Silk J. S. Dahl R. E. Ostapenko L. Kronhaus D. M. Phillips M. L. (2009). Fearful faces influence attentional control processes in anxious youth and adults. *Emotion* 9 855–864. 10.1037/a0017747 20001128

[B48] Lakes K. D. Swanson J. M. Riggs M. (2012). The reliability and validity of the English and Spanish Strengths and Weaknesses of ADHD and Normal Behavior Rating Scales in a preschool sample: continuum measures of hyperactivity and inattention. *J. Atten. Disord.* 16 510–516. 10.1177/1087054711413550 21807955 PMC3575190

[B49] Lewis M. D. Granic I. Lamm C. Zelazo P. D. Stieben J. Todd R. M. (2008). Changes in the neural bases of emotion regulation associated with clinical improvement in children with behavior problems. *Dev. Psychopathol.* 20 913–939. 10.1017/S0954579408000448 18606038

[B50] Luk G. Anderson J. A. Craik F. I. Grady C. Bialystok E. (2010). Distinct neural correlates for two types of inhibition in bilinguals: response inhibition versus interference suppression. *Brain Cogn.* 74 347–357. 10.1016/j.bandc.2010.09.004 20965635

[B51] Marx I. Domes G. Havenstein C. Berger C. Sczulze L. Herperts S. C. (2011). Enhanced emotional interference on working memory performance in adults with ADHD. *J. Biol. Psychiatry* 12 70–75. 10.3109/15622975.2011.599213 21905999

[B52] McDermott J. M. Perez-Edgar K. Henderson H. A. Chronis-Tuscano A. Pine D. S. Fox N. A. (2009). A history of childhood behavioral inhibition and enhanced response monitoring in adolescence are linked to clinical anxiety. *Biol. Psychiatry* 65 445–448. 10.1016/j.biopsych.2008.10.043 19108817 PMC2788124

[B53] Meule A. (2017). Reporting and interpreting working memory performance in n-back tasks. *Front. Psychol.* 8:352. 10.3389/fpsyg.2017.00352 28326058 PMC5339218

[B54] Miyake A. Friedman N. P. Emerson M. J. Witzki A. H. Howerter A. Wagar T. (2000). The unity and diversity of executive functions and their contributions to complex “frontal lobe” tasks: a latent variable analysis. *Cogn. Psychol.* 41 49–100. 10.1006/cogp.1999.0734 10945922

[B55] Morales J. Calvo A. Bialystok E. (2013). Working memory development in monolingual and bilingual children. *J. Exp. Child Psychol.* 114 187–202. 10.1016/j.jecp.2012.09.002 23059128 PMC3508395

[B56] Mueller S. C. Cromheeke S. Siugzdaite R. Boehler N. C. (2017). Evidence for the triadic model of adolescent brain development: cognitive load and task-relevance of emotion differentially affect adolescents and adults. *Dev. Cogn. Neurosci.* 26 91–100. 10.1016/j.dcn.2017.06.004 28688343 PMC6987860

[B57] Mueller S. C. Hardin M. G. Mogg K. Benson V. Bradley B. P. Reinholdt-Dunne M. L. (2012). The influence of emotional stimuli on attention orienting and inhibitory control in pediatric anxiety. *J. Child Psychol. Psychiatry* 53 856–863. 10.1111/j.1469-7610.2012.02541.x 22409260 PMC3427735

[B58] Nuske H. J. Hedley D. Woollacott A. Thomson P. Macari S. Dissanayake C. (2017). Developmental delays in emotion regulation strategies in preschoolers with autism. *Autism Res.* 10 1808–1822. 10.1002/aur.1827 28695672

[B59] Owen A. M. McMillan K. M. Laird A. R. Bullmore E. (2005). N-back working memory paradigm: a meta-analysis or normative functional neuroimaging. *Hum. Brain Mapp.* 25 46–59. 10.1002/hbm.20131 15846822 PMC6871745

[B60] Paap K. R. Greenberg Z. (2013). There is no coherent evidence for a bilingual advantage in executive processing. *Cogn. Psychol.* 66 232–258. 10.1016/j.cogpsych.2012.12.002 23370226

[B61] Paelecke-Habermann Y. Pohl J. Leplow B. (2005). Attention and executive functions in remitted major depressed patients. *J. Affect. Disord.* 89 125–135. 10.1016/j.jad.2005.09.006 16324752

[B62] Pool E. Brosch T. Delplanque S. Sander D. (2016). Attentional bias for positive emotional stimuli: a meta-analytic investigation. *Psychol. Bull.* 142 79–106. 10.1037/bul0000026 26390266

[B63] Poulin-Dubois D. Blaye A. Coutya J. Bialystok E. (2011). The effects of bilingualism on toddlers’ executive functioning. *J. Exp. Child Psychol.* 108 567–579. 10.1016/j.jecp.2010.10.009 21122877 PMC4346342

[B64] Prior A. MacWhinney B. (2010). A bilingual advantage in task switching. *Bilingualism* 13 253–262. 10.1017/S1366728909990526 PMC9724810 36479004

[B65] Raven J. C. Court J. H. Raven J. (1996). *Coloured Progressive Matrices.* London: H. K. Lewis.

[B66] Rose E. J. Ebmeier K. P. (2006). Pattern of impaired working memory during major depression. *J. Affect. Disord.* 90 149–161. 10.1016/j.jad.2005.11.003 16364451

[B67] Schmeichel B. J. Tang D. C. (2015). Individual differences in executive functioning and their relationship to emotional processes and responses. *Curr. Dir. Psychol. Sci.* 24 93–98. 10.1177/0963721414555178

[B68] Shackman J. E. Shackman A. J. Pollak S. D. (2007). Physical abuse amplifies attention to threat and increases anxiety in children. *Emotion* 7 838–852. 10.1037/1528-3542.7.4.838 18039053

[B69] Shields A. Cicchetti D. (1997). Emotion regulation among school-age children: the development and validation of a new criterion Q-sort scale. *Dev. Psychol.* 33 906–916. 10.1037/0012-1649.33.6.906 9383613

[B70] Shipstead Z. Harrison T. L. Engle R. W. (2015). Working memory capacity and the scope and control of attention. *Attent. Percept. Psychophys.* 77 1863–1880. 10.3758/s13414-015-0899-0 25911154

[B71] Stieben J. Lewis M. D. Granic I. Zelazo P. D. Segalowitz S. (2007). Neurophysiological mechanisms of emotion regulation for subtypes of externalizing children. *Dev. Psychopathol.* 19 455–480. 10.1017/S0954579407070228 17459179

[B72] Swanson J. Schuck S. Mann M. Carlson C. Hartman C. Sergeant J. (2001). *Over-identification of extreme behavior in the evaluation and diagnosis of ADHD/HKD.* Available at: http://www.adhd.net/SWAN_Paper.pdf

[B73] Thomas L. A. De Bellis M. D. Graham R. LaBar K. S. (2007). Development of emotional facial recognition in late childhood and adolescence. *Dev. Sci.* 10 547–558. 10.1111/j.1467-7687.2007.00614.x 17683341

[B74] Tottenham N. Tanaka J. Leon A. C. McCarry T. Nurse M. Hare T. A. (2009). The NimStim set of facial expressions: judgments from untrained research participants. *Psychiatry Res.* 168 242–249. 10.1016/j.psychres.2008.05.006 19564050 PMC3474329

[B75] Villemonteix T. Marx I. Septier M. Berger C. Hacker T. Bahadori S. (2017). Attentional control of emotional interference in children with ADHD and typically developing children: an emotional *N*-back study. *Psychiatry Res.* 254 1–7. 10.1016/j.psychres.2017.04.027 28437666

[B76] Vitiello V. E. Greenfield D. B. Munis P. George J. (2011). Cognitive flexibility, approaches to learning, and academic school readiness in Head Start Preschool Children. *Early Educ. Dev.* 3 388–410. 10.1080/10409289.2011.538366

[B77] Wechsler D. (1991). *The Wechsler intelligence scale for children*, 3rd Edn. San Antonio, TX: The Psychological Corporation.

[B78] Williams J. M. Watts F. N. MacLeod C. Mathews A. (1997). *Cognitive psychology and emotional disorders*, 2nd Edn. Chichester: John Wiley & Sons.

[B79] Wray E. Shashi V. Schoch K. Curtiss K. Hooper S. R. (2013). Discrepancies in parent and teacher ratings of social-behavioral functioning of children with chromosome 22q11.2 deletion syndrome: implications for assessment. *Am. J. Intell. Dev. Disab.* 118 339–352. 10.1352/1944-7558-118.5.339 24245728 PMC4124487

[B80] Young D. J. Levy F. Neilson C. M. Hay D. A. (2009). Attention Deficit Hyperactivity Disorder: a Rasch analysis of the SWAN Rating Scale. *Child Psychiatry Hum. Dev.* 40 543–559. 10.1007/s10578-009-0143-z 19455417

[B81] Zelazo P. D. Carlson S. M. (2012). Hot and cool executive function in childhood and adolescence: development and plasticity. *Child Dev. Perspect.* 6 354–360. 10.1111/j.1750-8606.2012.00246.x

[B82] Zelazo P. D. Cunningham W. A. (2007). “Executive function: Mechanisms underlying emotion regulation,” in *Handbook of Emotion Regulation*, ed. Gross J. J. (New York, NY: Guilford), 135–158.

